# A Physiological Study

**Published:** 1889-07-13

**Authors:** 


### The Hospital

A Weekly Institutional Journal of

**Science, Medicine, Nursing, and Philanthropy.**

*For Hospitals, Asylums, Medical Practitioners, Students, Nurses, Families, Parishes, Congregations, and the Charitable Public.*

Vol. VI. No. 146.] [July 13, 1889.

### A Physiological Sunday.

For good or for ill, physiology and physiologists are much with us in these times; and they ask and obtain a large share of public attention. By many people the voice of the physician is more readily listened to than the voice of the prophet, and a physiological textbook is regarded as of much higher authority than the Bible. This arises to a large extent from the natural limitations of the human mind. Not a few persons fail to understand that things may be diverse without being opposed to each other, and that a man may be a foreigner without being an enemy. Untrained minds, and sometimes minds that are, at least in a measure, trained, love sharp contrasts. For them a thing must be either white or black, good or bad, true or false. Because religion differs in some respects from science, one or other of them must be unworthy of credence, they think. But science cannot be unworthy of credence, since it deals with nothing but hard facts which we can see and handle, or, at least, touch. Therefore, of course, it is religion that must be politely bowed out.

Now, this is not really so in fact. It is, perhaps, quite true that one man may have naturally much more aptitude for religion than another, just as one shall have more aptitude for mathematics and another for music. In saying this we are not asserting that religion is merely a special faculty natural to certain individuals, but wanting in others. On the contrary, science seems to show that religion is natural to man as man all the world over. But it still remains true that some men are, by constitution, much more inclined to religion and much more ready to obey its teachings than are others. If, however, it be true, as it undoubtedly is, that religion is natural to man as man, then it cannot but be unscientific to treat man as if he were not a religious being, to ignore in fact, one of the fundamental constituents of his nature. The really scientific physiologist, in summing up the facts of man's constitution, physical and mental, and in giving a practical estimate of man and his needs and prospects, must take in all the facts that bear upon his religious nature and necessities, as well as every other fact that is essential to the complete conception of the perfect human creature.

Starting from this standpoint, what would an unprejudiced physiologist demand in a perfect Sunday? He would demand rest undoubtedly. The value of one day's rest in seven is so universally allowed that it would be an unpardonable waste of time to argue the subject afresh. But what is rest? To a man who works hard six days out of seven, is mere idleness and do-nothingness the best form of rest, or is it in any sense perfect rest at all? In cases where men have to perform exceedingly exhausting physical or mental labour during the week, it is not improbable that a Sunday spent in doing absolutely nothing at all may be very profitable. It is stated that one of the busiest modistes in Paris invariably spends Sunday in bed. Many people have heard of the poor washerwoman who, after toiling almost day and night for seventy years, came to her last day of life. She was quite happy at the prospect of death; and the consideration which gave her most happiness was this, that she was "going to do nothing for ever and ever." But the ordinary experience of men and women is not of this extreme kind. The average man in these times undoubtedly works hard. He puts his best energies into his business from morn till night, and from the beginning to the end of the week. On Saturday he is tired, thoroughly tired; but he is not usually so exhausted that, like a horse who has been "winded," he could not move another step to save his life. He needs rest, but the rest that is most suitable for him is not the rest of absolute do-nothingness, but the completest change he can get from strain to ease, from the narrow outlook of the moment to the broader one of all time and all life, from conflict with his fellows to peaceful agreement with them, from care and anxiety to the entire abandonment of care.

This kind of rest the religious man usually obtains in connection with the Sunday services of his church or in some kind of religious work. It cannot be doubted that so far as the rational and healthy use of the Sunday is concerned, the religious man has a decided advantage over every other class. There are, of course, ways of spending Sunday which are perfectly well known, and which are very far from being similar to the way which religion would prescribe. Many town men lounge about from morning till night; often not even washing and dressing themselves until mid-day. Then they will go out, and after a more or less considerable walk, call at some place of entertainment and drink beer or spirits. Now, we are not urging Sunday closing upon readers; but, since the truth ought always to be spoken, we venture to affirm that no physiologist would commend such a way of spending the Sunday as that. It is altogether too idle, too purposeless, too poor in intellectual exertion or pleasure to commend itself to the medical mind. If we were discussing the question from a religious point of view, we should speak much more strongly. But from the standpoint of a rational physiology the way in which many British workman and others spend their Sunday is as senseless and as profitless as it can well be.

The familiar proposal to open museums, botanical gardens, and other such places of recreation on the Sunday has much in its favour. The only objection to it seems to be that many officials and servants would thereby be compelled to work seven days a week instead of six. That difficulty can, however, be got over to some extent by alternations of attendance. There is no doubt very much to be learnt and a great deal of pleasure to be found in sight-seeing and open-air recreation of this kind. The idea of recreation should be one of the characteristic notes of Sunday; and so long as the recreation is harmless, conducive to health, and not unworthy of rational creatures, there should be a very wide margin of permitted and encouraged recreations. Dr. Farre—who as a medical authority had few equals, and perhaps no superior—gave his marked approval to the religious way of spending the Sunday. He held that, as a matter of physiology, it could be shown that the one day's rest in seven was absolutely indispensable to health; and he considered that the use of it as a day of "holy" rest was not only the highest in a moral sense, but the most rational to which it could be put. The Sabbath, he said, when so employed was "a source of renewed vigour to the mind, and through the mind to the body," and "an additional spring of life."

London cannot be congratulated, either from a physiological or a moral point of view, on the way in which many of its inhabitants spend their Sunday. It is said that even ten or twelve years ago there were as many shops open as would have extended in a straight line for a distance of sixty miles; whilst the public-houses and beershops would have stretched from Charing Cross to Portsmouth, a journey of seventy-three miles. All this implies an amount of Sunday labour and Sunday confinement indoors which is appalling. Putting out of sight the religious question altogether, the ruin to health and the consequent ruin to morals is sufficient to bring the most heedless and sceptical to their senses. No reasonable man can possibly defend the present state of things or desire its continuance. It is stated by those who know, that no improvement need be looked for except as the result of legislation. Then legislation should certainly be invoked. If, as a matter of public well-being, we think it desirable and imperative to enact sanitary laws, and to include within their scope such questions as compulsory vaccination, it is surely within the right and duty of Parliament to put its foot down firmly against Sunday trading, and that not only in the interests of shop-assistants but of their employers also. Seven days' labour in every week is destruction, and that in no long time, to all who practise it. If we were, as we think we are, a highly rational and courageous people, we should no more think of permitting a quarter of our town population to work seven days a week than we should of allowing them to escape the Vaccination Act, or to run about the streets covered with smallpox.

